# Speech acts in the Dutch COVID-19 Press Conferences

**DOI:** 10.1007/s10579-022-09602-7

**Published:** 2022-07-19

**Authors:** Daan Schueler, Maarten Marx

**Affiliations:** IRLab, Informatics Institute, University of Amsterdam, Amsterdam, The Netherlands

**Keywords:** Dutch COVID-19 Press Conferences, Speech act theory, Text classification

## Abstract

An open source corpus of all Dutch COVID-19 Press Conferences with sentences annotated on the basis of John Searle’s Speech Act taxonomy was created. It contains all 58 press conferences held between March 6 2020 and April 20 2021 and has 9.441 manually annotated sentences. Speech acts were annotated in a consistent manner, with a Krippendorff’s alpha of .71. The corpus is easy to use and rich in metadata, with lexical, syntactic, discourse (speaker, question or answer) features and information on the type of regulations being present. We analyse the press conferences in terms of speech act usage, giving insight into the use of speech acts over time, the relation of speech act usage to real world phenomena, the general structure of the press conferences and the division of roles between speakers. Relations were found between speech act usage and the type of press conference (i.e. easing, tightening or neutral) as well as the number of hospital admissions. Speech act classes showed preferred locations within the press conferences, indicating a general structure. Distinct roles between speakers were identified. We also investigate the use of our set of labelled sentences for training a speech act classifier and achieve a reasonable accuracy of .73 and a mean reciprocal rank of .74 with the state of the art transformer RoBERTa model.

**Supplementary Information:**

The online version of this article contains supplementary material available 10.1007/s10579-022-09602-7.

## Introduction

In December 2019, the first case of COVID-19 (Coronavirus) was identified in Wuhan. On the 30th of January 2020 the World Health Organisation (WHO) declared the virus outbreak to be a public health emergency of international concern. In February 2020, the Netherlands’ first case was identified. For the Dutch, this was the start of their fight against the pandemic. To exit these troubled times, it was deemed necessary that people change their behaviour. The Dutch government communicates information and new regulations regarding the Coronavirus disease by means of press conferences (PCs). One of the main goals of the PCs is to address the behavioural changes necessary to lower the number of infections. These press conferences have become a characteristic element of the Coronavirus pandemic, warranting thorough scientific analysis from a wide range of fields, psychology, communication science, epidemiology, social sciences, linguistics.

The Corona PCs had a direct impact on a huge number of people. The top 10 most watched television programs in 2020 were Corona PCs, with number of viewers ranging from 8.6 to 4.5 million (of a population of 18 million, and with an every year top 10 program at New Year’s Eve having 4M viewers).

This paper has three main aims. First, creating a rich, open and easy to use corpus of all Dutch COVID-19 Press Conferences and providing each sentence with lexical syntactic, and discourse information. Second, analysing the press conferences using speech act theory and relating the used speech acts to the course of the pandemic in the Netherlands. Third, testing whether the task of labelling sentences by speech acts can be automatized using supervised machine learning.

This research analyses the press conferences in terms of John Searle’s Speech Acts: Assertives, Directives, Commissives, Expressives and Declaratives (Searle, 1985), at the level of sentences. A sentence can contain zero, one, or multiple speech acts. Analysing these speech acts in the press conferences can give a variety of insights. For instance, filtering the dataset on Declaratives will show the amount as well as the course of the proclamation of regulations. The proportion of Modest and Strong Directives gives insight into the approach of the governmental representatives when it comes to steering citizens’ behaviour. This in turn could be compared to the proportion of Modest Directives and Strong Directives of other countries, which possibly impose a different approach. Filtering the data on Commissives will indicate the intentions of the speakers and the Expressives can give an indication of the expressed emotional involvement. Additionally we can use the speech acts to structure the press conferences and to find differentiating roles played by the speakers.

Our main research question is: *How do speech acts manifest themselves in the Dutch COVID-19 Press Conferences?*. This research question is divided into two sub questions:

SQ 1. How well can the speech acts be identified in the Dutch COVID-19 Press Conferences?

SQ 2. How can the Dutch COVID-19 Press Conferences be described in terms of these speech acts?

- SQ 2.1 What is the overall speech act distribution of the Press Conferences?
- SQ 2.2 To what extent does the distribution of the speech acts change over time?
- SQ 2.3 How do the speech act distributions relate to Covid phenomena, like the number of infections or the tightening or easing of measures?
- SQ 2.4 Do speech acts classes have a preferred location in the press conferences and does this differ for press conferences in which regulations are eased or
- SQ 2.5 Is there a difference in speech act usage between Prime Minister Mark Rutte and Minister of Health, Welfare and Sport Hugo de Jonge?

We will also find out whether machine learning can speed up the annotation process.

The paper is organized as follows. Section 2 recalls Searle’s Speech Act theory and describes the speech acts we use, and Sect. 3 contains related work. Section 4 describes the data, used methods and the annotation process. Section 5 contains the results and we conclude in sect. 6. The Appendix contains the used annotation protocol. All data (both raw and processed and annotated), and data gathering and analysis scripts are permanently available at the Dutch Scientific Data Repository DANS at URL https://doi.org/10.17026/dans-2af-rwmr.

*Main contributions* Our contribution is twofold, following the research questions. First, we make the Dutch Corona Press Conferences readily available for research in a well structured, easy to use format with relevant metadata. The annotation of sentences with speech acts is of good quality, witnessed by a Krippendorf $\alpha$ score of .71 and as well by the fact that we can train a speech act classifier on the data with an accuracy of .73. Second, we show that the use of speech acts is related to the strength of the pandemic, the type of measurements being announced, and the role of the speaker. Of all sentences spoken in the Corona PCs, over one third is classified as a non-assertive speech act. Speech acts have rather stable, preferred places in the PCs.

## Searle’s speech act theory

In the mid 20th century, language philosopher John Langshaw Austin (1975) expressed his ideas on what he called *performative utterances*, published under the title *How to Do Things with Words*. . Going further than Austin’s work, John Searle provided a general framework for a theory of speech acts as well as a richer and more detailed specification and structure of the speech acts (Searle, 1985). Searle’s five classes of Speech Acts consist of *Assertives*, *Directives*, *Commissives*, *Expressives* and *Declaratives*. We briefly recall what these are, and provide an example from our corpus of Press Conferences.

*Assertives* With an Assertive, the speaker wants to commit the hearer to his belief on how things are in the world. The speaker says something is being the case, or will state a representation of reality. The statement can be assessed to be true or false Searle (1985). Example: “De instroom en het aantal corona patiënten in de ziekenhuizen vlakken nu af.” (*The number of Corona patients in the hospitals is decreasing.*)

*Directives* Directives are attempts by the speaker to try to get the hearer to do something, referring to future acts. They are obeyed or disobeyed and can be either modest or strong. Example (modest directive): “Daarom roep ik iedereen op om wat vaker ’s ochtends de boodschappen te doen, want dan is het een stuk rustiger in de winkels.” (*For this reason, I ask everyone to shop in the morning, as the shops are then much less crowded.* An example of a Strong Directive is: “Die mensen, die dus voor hun werk op pad zijn, moeten dan ook een werkgeversverklaring bij zich hebben.” (*Those people, being on the road for work related purposes, need to carry a statement by theor employer.*)

*Commissives* Commissives commit the speaker to some action in the future. Example: “En wie er niet aan voldoet en bijvoorbeeld toch klanten toelaat in de winkel, die wordt gesloten.” (*Those who do not comply, and for instance admit customers into their shops, are closed.*)

*Expressives* With Expressives, the speaker expresses his psychological attitudes and emotions towards the state of affairs, stating what the speaker feels. Example: “En daar heb ik ook zelf de afgelopen dagen enorm mee geworsteld.” (*Me myself has struggled with this enormously the past few days.*)

*Declaratives* Declaratives change the world by verbally stating the change. For a Declarative to be performed successfully, the speaker must have some contextual privileges that allow her to declare the change. The status of the speaker and the hearer as well as their social position come into play ( Fotion (2000), p. 52). Example: “Vanaf woensdag 28 april mogen de buitenterrassen onder voorwaarden weer open van 12 tot 6 uur s ’middags.” (*Starting Wednesday April 28, outside terraces are allowed to be open, under restrictions, from 12 to 6 in the afternoon.*)

Sentences need not express a speech act, and they can also express multiple speech acts. We have formalised these six (we distinguish between the modest and the strong Directives) different speech acts in the annotation guidelines present in Online Appendix B.

## Related work

The construct of speech acts as defined by Searle has been addressed and described by many, e.g., Fotion (2000) and Smith (2003). The construct is also criticized. For example, Love (1999) and Rajagopalan (2000) highlight contradictory states of affairs. The setting of this research however, is not language philosophical, but applicational: we want to use Searle’s taxonomy of Speech Acts to structure a set of highly influential texts.

De Felice et al. (2013) report on the process of manual annotation of speech acts in a corpus of business e-mails. They did this in the context of the PROBE project (Pragmatics of Business English). The aim of manually annotating this corpus was to shed light on the speech acts’ linguistic and discourse structures in business e-mails and to assess how well the theoretical constructs relate to real world data. The speech act categories used were focused on requests, commitments, expressions of feeling and statements. These translate to *Directives*, *Commissives*, *Expressives* and *Assertives* in Searle’s taxonomy.

Forsythand and Martell (2007) indicate a gap in annotated chat corpora available to the broader research community. The purpose of their research was to build a chat corpus that could be used to develop more complex NLP applications. Their corpus was tagged with lexical information, syntactic information and discourse (classification) information.

Moldovan et al. (2011) use supervised machine learning methods to classify online chat posts into speech act categories. They used the annotated Linguistic Data Consortium (LDC) chat corpus constructed by Forsythand and Martell (2007). They used the first two to six words and their Part-Of-Speech (POS) tag as features. Their research supports the hypothesis that the first few tokens/words in a chat post are very predictive of the post’s speech act category.

Subramanian et al. (2019) study pragmatics in political campaign text. For each utterance, they analyse the corresponding speech act and the target of the utterance. They present an annotated corpus of media releases and speech transcripts from the 2016 Australian election cycle. They study the effect of jointly modeling the speech act and target referent of each utterance to determine the intent of every utterance and, in turn, automatically extract pledges (Commissives) made by politicians from campaign speeches and press releases.

Qadir and Riloff (2011) study message board forums. These forums contain expository sentences that present factual information and conversational sentences that present communicative acts. The goal of their study is to create a sentence classifier that identifies if a sentence contains one of four speech acts: *Commissives*, *Directives*, *Expressives* and *Representatives* (equivalent of *Assertives*). Declarations were virtually not present in their data, therefore this class was disregarded. They achieved good results for the identification of *Directives* and *Expressives*, but found that *Assertives* and *Commissives* were more difficult to identify.

De Felice and Deane (2012) developed a computational model for automated speech act identification in the context of the TOEIC writing e-mail task. TOEIC tests are designed to measure English language skills in a business context. The researchers tested their model on the TOEIC responses, achieving up to 79.28% accuracy. Their classification focused on subclasses of requests, orders and commitments, which translate to *Modest Directives*, *Strong Directives* and *Commissives* in Searle’s taxonomy.

Hacquebord (2017) investigates multiple dialogue act recognition methods in the setting of conversational agents and determines what methods do and do not work. Additionally, an alternative approach to dialogue act recognition is proposed to counter some of the identified issues. For this research, a more extensive annotation scheme was used, namely 42 clustered SWBD-DAMSL dialogue act tags.

## Data and methods

This section addresses the data and methods that are used. The first subsection describes the collection of the press conferences, followed by the methods used to create the corpus. We then discuss the annotation process and protocol, and how we measure inter-rater reliability. In the third subsection, we review extra metadata relating the PCs to the Covid reality.

### Description of the data

#### Collection of the data

The press conferences were broadcasted live on public television and later transcribed and published on the government’s official website (www.rijksoverheid.nl). A total of 60 press conferences were collected. These press conferences were published between March 6th 2020 and April 20th 2021. One of the collected PCs was specifically dedicated to answering questions of children. This PC was very informal and had two additional hosts that would help the communication between the children and the governmental representatives. It was considered an outlier and was therefore removed. The PC on the 23rd of February 2021 was published twice and also removed, resulting in a final corpus of 58 PCs.

#### Creation of the corpus

The transcribed PCs were published in HTML. For each PC, the date and the transcript were extracted and all parts of the spoken text was assigned to its speaker. The text was split into sentences using NLTK (Bird et al., 2009). Furthermore, the following metadata at sentence level was added: its rank in the PC, the date of the PC, the speaker, whether the speaker was a minister or journalist, if the sentence was part of the introductory statement or the QA session, if the sentence was part of a question by a journalist, or an answer to a question, and if so, to which question. Finally, SpaCy was used tokenization, lemmatization, part of speech tagging and chunk parsing.

#### Brief overview of the corpus

**Fig. 1:**
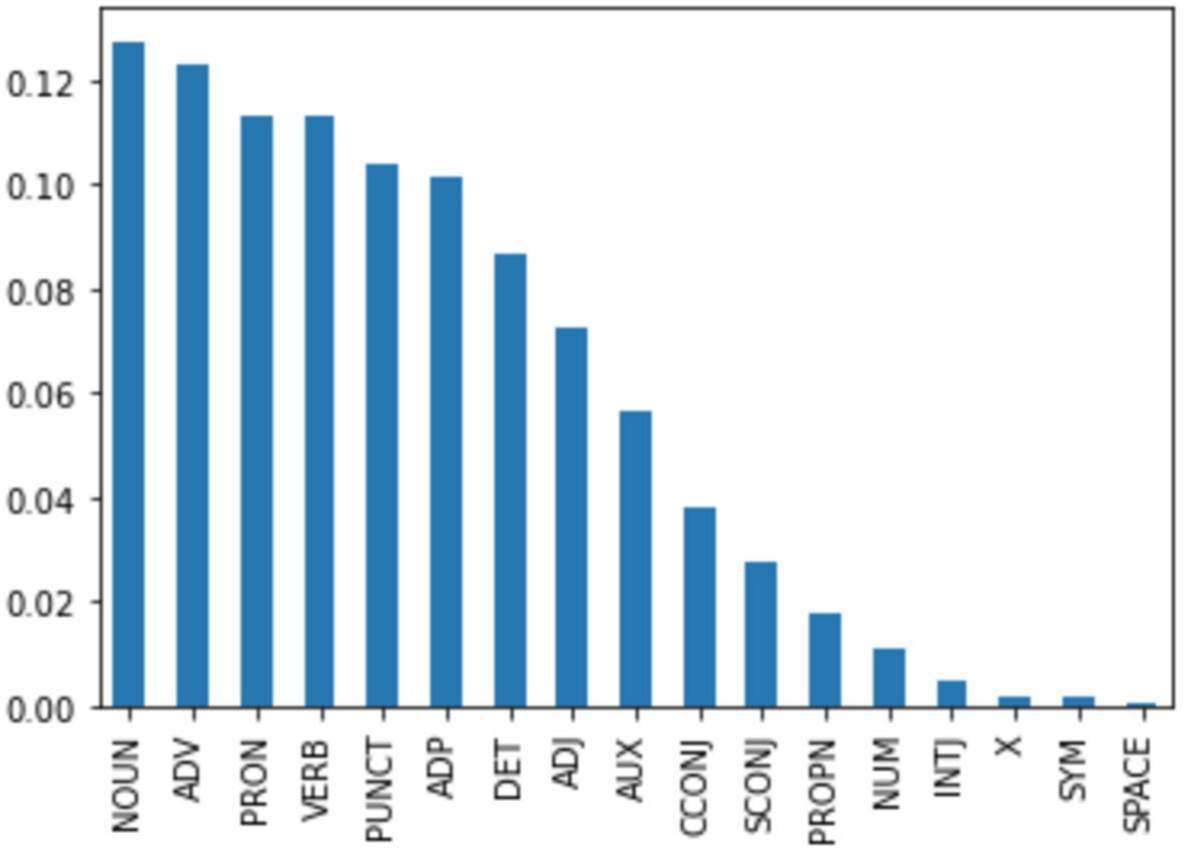
Distribution of the part of speech tags (tagged with SpaCy) in the press conference corpus

The corpus thus consists of 58 press conferences held between March 6 2020 and April 20 2021. In 14 of these, Covid measures were tightened, and in 7 easened. The corpus contains 5.548 paragraphs, 29.409 sentences, 528.703 tokens, 15.431 unique words, and 11.083 unique lemmas. It contains 2.678 question-answer pairs. A question is on average 2 sentences long, and an answer 7 sentences. There are 183 identified speakers, of which 11 are government officials and 172 journalists (asking questions). Figure 1 contains the proportions of the most frequent parts of speech.

### Annotating speech acts

We manually labelled individual sentences with zero, one, or more speech acts. The full corpus consists of almost 30K sentences. We had to make a selection, which we now motivate. Of these sentences 5.5K sentences were spoken by journalists. As the interest of this research is in the use of speech acts by governmental representatives, the sentences spoken by journalists were disregarded in the annotation process. Of the 24K sentences that remained, which were all spoken by governmental representatives, 5.749 sentences were used in the introductory statements of the press conferences, and the rest were answers to questions of journalists.

However, when these PCs were broadcasted live on television, the broadcaster only showed the introductory statements and only a few questions and answers. Therefore, the same was done in the annotation process. As the aim of the PCs is to communicate information and new regulations regarding COVID-19 to the members of the public, the annotation process was also aimed on the sentences that were actually heard by the public. The cut off point by the broadcasters was somewhat arbitrarily set at the first ten responses given to questions. This resulted in the manual annotation of a corpus consisting of 9.441 sentences.

We used the annotation tool Prodigy (https://prodi.gy). Prodigy provides a simple interface in which the annotator sees a sentence and selects the applicable speech acts. The use of Prodigy considerably sped up the annotation process, allowing the annotators to annotate around 200 sentences per hour. Yet, the annotation process was a time consuming task, taking up roughly 50 hours of effective annotation time.

#### Annotation protocol

The corpus was annotated by two annotators. To ensure consistent annotation, also between annotators, an annotation protocol was constructed, partly based on Weisser (2014). The constructed protocol was in line with Searle’s theoretical taxonomy of Speech Acts. As Searle indicates a difference between a Modest Directive and a Strong Directive, this distinction was also made in the annotation protocol. Zero, one or more Speech Act classes were assigned to each sentence. Sentences that were split incorrectly, or were unfinished sentences due to the spoken nature of the sentences were rejected/ignored (e.g. *Dus wij naar streven is om de...*). This was the case for only 37 sentences. The full annotation protocol can be found in “Appendix 1”.

#### Annotation quality

To evaluate the quality of the annotated corpus, the inter-rater reliability score was computed using Krippendorff’s $\alpha$ (Krippendorff, 2011). To calculate Krippendorff’s $\alpha$, the first three out of the 58 press conferences were annotated by both annotators (i.e. 622 out of 9.441 sentences). Krippendorff’s $\alpha$ is defined as $1- D_o/D_e$, with $D_o$ the observed disagreement among the values that were assigned to the sentences and $D_e$ the disagreement one would expect when the assigned value is attributable to chance rather than to the properties of these sentences. The minimum $\alpha$ for a corpus to be acceptable is usually taken as .60. Our annotations can consist of multiple labels. An annotation was only seen as an agreement when both annotators identified the same set of speech acts for a sentence (including the empty set). This meant that if *Annotator A* identified a sentence to be Assertive and Commissive while *Annotator B* identified the sentence to be Commissive, this sentence would be seen as a disagreement even though both annotators identified the Commissive in the sentence. This strictest way of measuring inter-rater reliability resulted in a score of $\alpha =.60$.

The main discrepancy between coders was found in the multi-labeling of utterances. When looking only at the 537 (of the 622) utterances that were single labeled by both annotators, $\alpha$ was .71. Thus, an important difficulty were the multi labeled sentences. In total, there were 17 different speech act combinations used in the control sentences (out of the possible $2^6=64$). Most of these combinations involved Assertives. Fotion (2000) addressed the presence of Assertives in indirect or implicit speech acts and the difficulties accompanying these utterances. As indirect speech acts were often formulated in an Assertive way, Assertives often accompanied other speech acts. Fotion (2000) gives the example “You’re standing on my foot.”, which literally is an assertive, but implicitly a directive (to remove your foot from mine). Following this example, whether or not this utterance should be annotated as an Assertive or an Assertive Directive was the main discrepancy between the annotators. Because the aim of the utterance is Directive, it can be argued that such a sentence should not be classified as an Assertive as well as a Directive, but a Directive only. After resolving this conflict, i.e. removing Assertives in this type of multi-classified sentences, the inter-rater reliability score improved to $\alpha =.70$.

The take-away from this was that the annotation of Assertives in combination with other speech acts was a point of attention. The protocol was adjusted and the corpus was annotated using these improved annotation guidelines. The annotations of this type of Assertives were then consistently applied on the corpus, with annotators continuously consulting each other when they were uncertain on the classification of an utterance.

### Additional context to the press conferences

The speech act distributions were related to two real world phenomena. First, the type of press conference was derived from reading the introductory statement for each press conference. Press conferences in which additional measures were declared or in which existing measures were tightened, were marked as tightening press conferences. Out of the annotated press conferences, 24% were tightening. Press conferences in which existing measures were eased, were marked as easing press conferences (14%). Press conferences in which no measures were tightened or eased, i.e. measures were continued, were marked as neutral press conferences (62%).

**Fig. 2:**
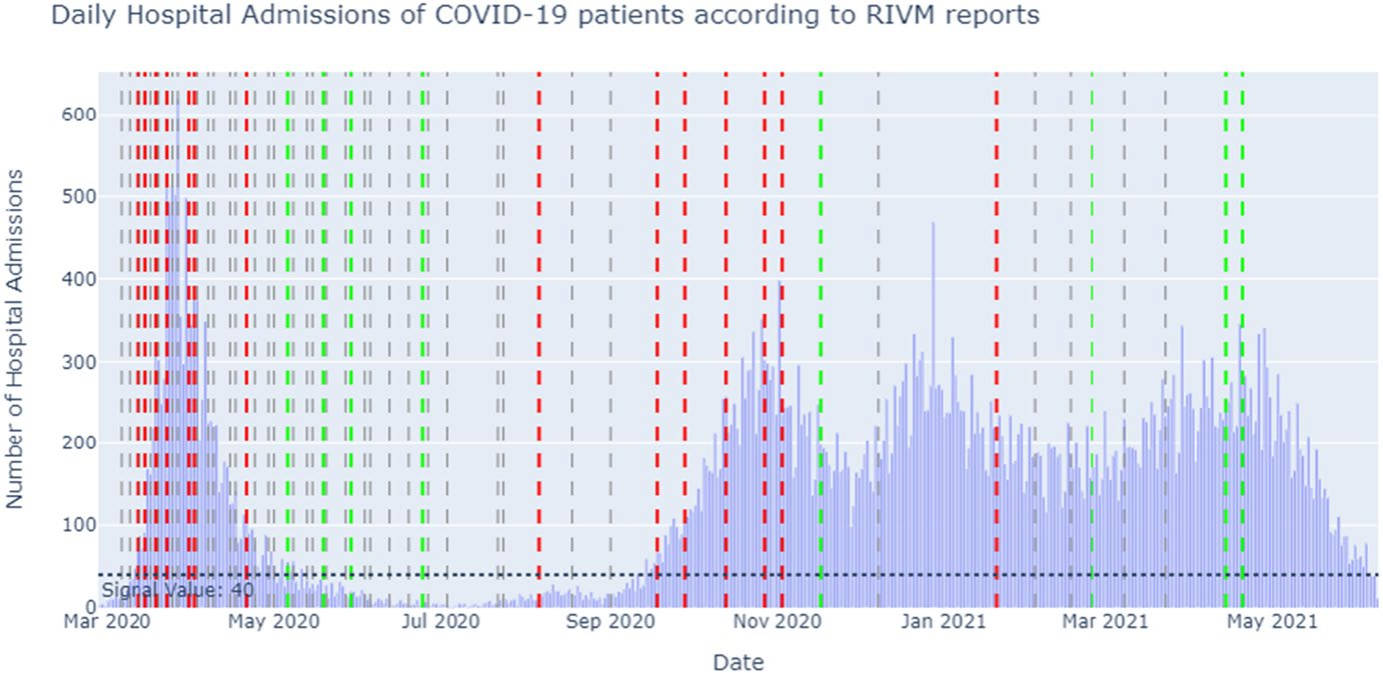
The daily hospital admissions between March 2020 and June 2021 according to RIVM reports together with all press conferences and their type. The red dotted lines are tightening, the green dotted lines are easing and the grey dotted lines are neutral press conferences

We also related speech act usage to the number of daily hospital admissions of COVID-19 patients, as published by the Dutch RIVM. Figure 2 shows the number of hospital admissions, combined with the type of press conference. The red, green and grey dotted lines indicate the presence of a tightening, easening and neutral PC, respectively. The figure shows a general trend of tightening press conferences at times of increasing or high hospital admissions and easing press conferences in times of decreasing or low hospital admissions. Section 5.2.3 elaborates on these topics and relates them to speech act usage.

## Results

This section addresses the research questions. We start with assessing the quality of the speech act annotation. Then we describe the press conferences in terms of the speech acts, following the subquestions. And finally we evaluate how well a machine learned speech act classifier can help in reducing the annotation time and costs.

### Identifying speech acts in the Dutch COVID-19 press conferences

Section 4.2.2 described that the inter-rater reliability as measured by Krippendorff’s $\alpha$ was .71 for the single labelled sentences and .70 for all sentences. This is generally considered a viable score.

There are some points of attention, when annotating speech acts. The difficulty of implicit speech acts has already been covered above. Furthermore, the correct classification can depend on context and world knowledge not present in the sentence, and not even in the complete document. Consider for example, the sentence:

“Je moet je echt wel houden aan datgene wat ook in de bijsluiter staat, waar ook de EMA zijn uitspraak over heeft gedaan.” (*You must stick to the package leaflet [of the Corona vaccine], as also pointed out by the EMA.*)

From the surrounding sentences, we can infer that the sentence is about the package leaflet of the Corona vaccine. The sentence seems to indicate that the speaker insists the listener to do something, namely follow the prescriptions of the Corona vaccine. Without any context, one would classify this as a Directive. However, the speaker does not refer to anything the listener has any influence on. Namely, the speaker refers to the government’s policy on vaccination. The sentence indeed is a statement in which the speaker addresses the importance of sticking to the prescription of the vaccines, for which he himself is responsible. Viewed as such, the sentence could be taken as an, albeit very implicit (but after all, this is a politician speaking) Commissive.

### Describing the press conferences in terms of speech acts

We now analyse the press conferences in terms of the annotated speech acts, covering five topics. First, we look at the overall distribution of the speech acts. Then we see how this distribution changes over time during the pandemic. Third, we relate the found distributions to the severity of the pandemic and to the main message of the PC. Fourth, we look whether certain speech acts have a preferred location within the press conferences. Finally, we look whether the different cabinet roles (Prime Minister and Minister of Health, Welfare and Sport) of the two speakers in the PCs is reflected in a different usage of speech acts.

Before diving into these five topics, it is important to note that because we are dealing with multi-labeled sentences, all distributions are normalized based on the number of annotations, not on the number of annotated sentences.

#### Overall speech act distribution

**Fig. 3:**
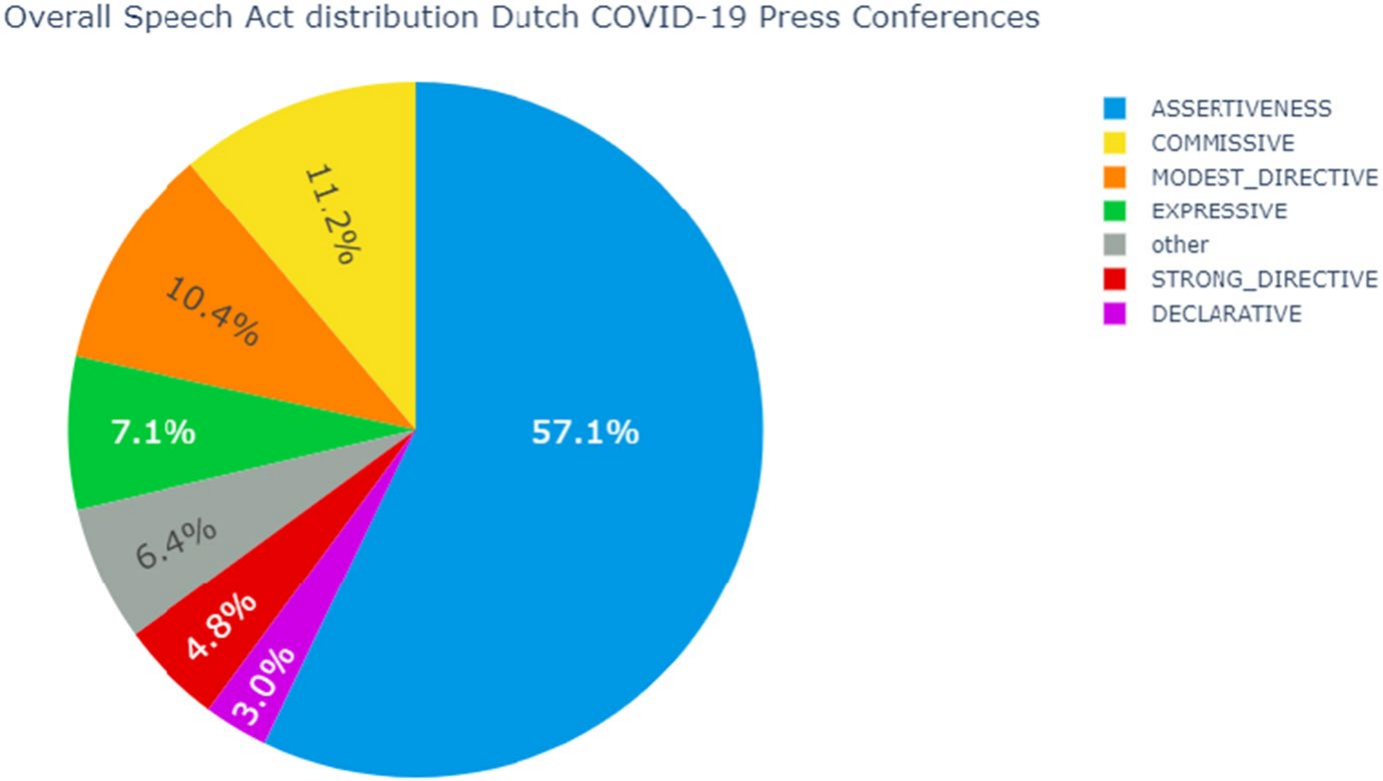
The distribution of speech act usage in all Dutch COVID-19 Press Conferences

The overall speech act distribution is given in Fig. 3. As expected, the majority of annotations are Assertive. Taking the Modest and Strong Directives together, the Directives form the second largest speech act class and Modest Directives are used more than Strong Directives. The governmental representatives prefer requesting the people for their cooperation and pleading for compliance with regulations as opposed to ordering and commanding people to show certain behaviour. In a press conference on the 8th of May 2020, Prime Minister Mark Rutte expressed his view on his position: “Ik wil helemaal niet de baas spelen hier, dat ben ik ook helemaal niet.” Which translates to him saying he is not the boss, nor does he want to be.

The third largest speech act class is the Commissive, followed by the Expressive. Roughly six percent of the sentences were not assigned a speech act, as they did not belong to any of the speech act classes.

The smallest and therefore also notable speech act class in this distribution graph is the Declarative. In total, only three percent of all annotations were Declaratives. Declaratives refer to those utterances in which the speaker needs some contextual privileges to declare change by verbally stating it. In the context of the press conferences, these utterances have to do with easing, tightening and extending measures. As opposed to what one might expect, these utterances reflect only a small portion, namely three percent, of the annotations in the press conferences.

#### Change of speech act distribution over time

**Fig. 4:**
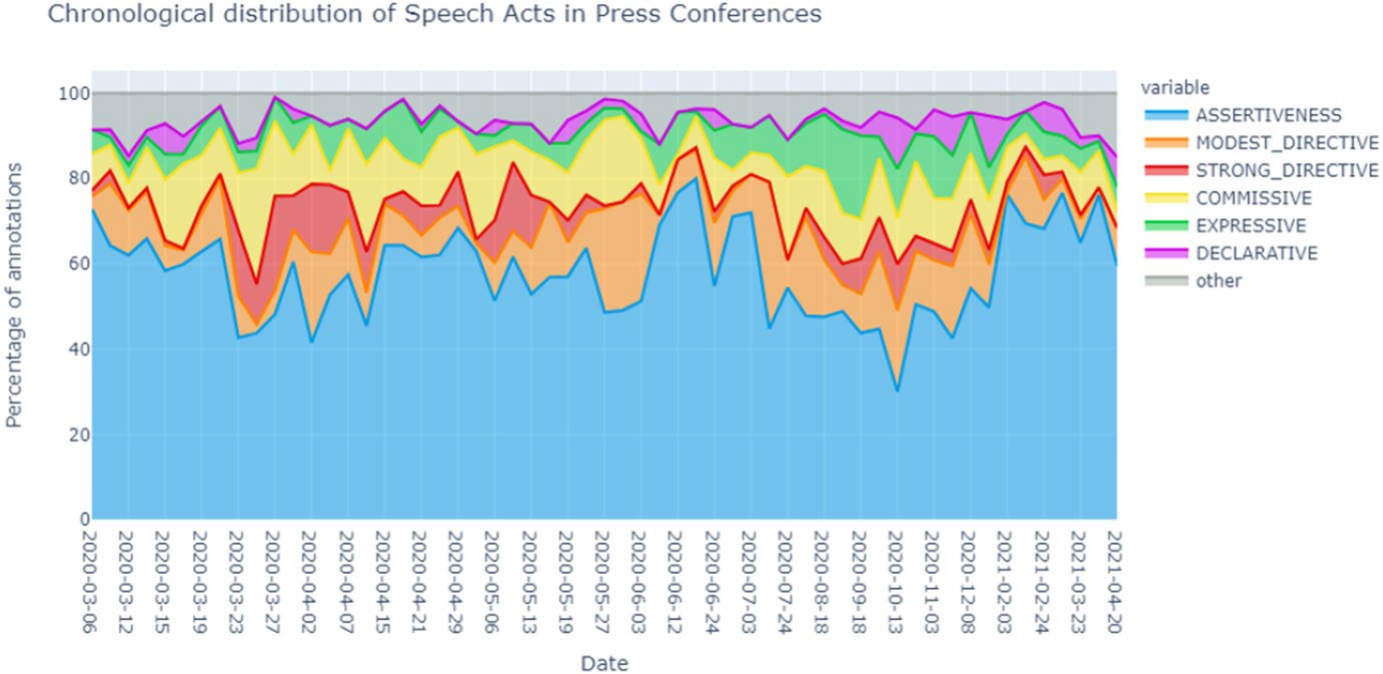
Chronological streamgraph showing the distribution of speech acts in the individual Dutch COVID-19 Press Conferences

Figure 4 shows how the distribution of the speech acts per PC. This figure can be seen as a sequence of pie charts. The x-axis is chronological but not proportional with time, as PCs were much more frequent in the beginning of the pandemic. Figure 11 in the Appendix presents the same information as a stacked bar chart, allowing an easier comparison of specific PCs.

The figure shows that the distribution varies quite a lot over time. In general, Assertives remain dominant, peaking on the 19th of June 2020 and dropping on the 13th of October 2020. Modest Directives are often present. There is a peak in Modest Directives in the end of May 2020 and in the end of July 2020. Then, they are quite consistently present in the months August through December 2020, lessening its presence a bit from the end of January to April 2021. Strong Directives are less consistently present. They were mostly present from the end of March 2020 to June 2020, with its peak in the beginning of April 2020. They were least present in the summer of 2020, regaining its presence in the fall of 2020. Commissives and Expressives are quite consistently present but also peak in certain periods. Finally, again, we see the notably minimal presence of the Declaratives.

The next subsection provides plausible explanations for some of these changes.

#### Speech act distributions and real world phenomena

Now that we have seen that speech act distributions change over time, it is interesting to find out if real world phenomena are responsible for these fluctuations. In this section we will look at two related phenomena. First, we will consider whether a press conference eases or tightens regulations or is neutral in thi aspect. Second, we look at the number of daily hospital admissions of COVID-19 patients.

##### Type of press conference

**Fig. 5:**
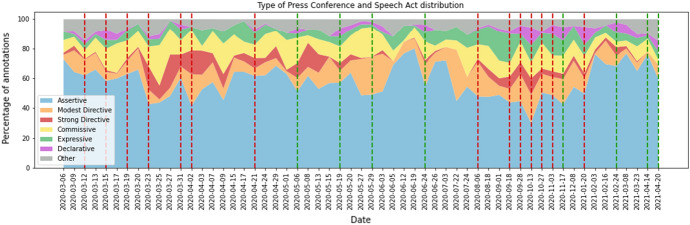
The type of press conference in combination with the speech act distributions. Tightening press conferences are marked with a red dotted line. Easing press conferences are marked with a green dotted line. The remaining press conferences are neutral press conferences

The first related phenomenon we are going to look at is the characterization of the press conferences in terms of easing or tightening regulations. In Figure 5, press conferences in which additional measures were declared or existing measures were tightened are marked with a red dotted line, and those in which regulations were eased with a green dotted line. The remaining press conferences can be seen as neutral press conferences, as in these press conferences regulations were not eased or tightened.

Looking at all dotted lines in general, most of the Declaratives overlap with the dotted lines. This means that the Declaratives are over represented in press conferences in which regulations are eased or tightened, as expected.

We expect the same over representation for the Modest and Strong Directives, and the opposite one for the Assertives: the focus of neutral press conferences is to inform people on the current state of affairs. For the Expressives and Commissives, we expect no difference between the two types of PCs.

Table 1 contains for the two groups of press conferences, the proportions of sentences labelled with each speech act, and whether that difference is significant. We see that indeed the intuitive expectation backed up by Fig. 5, is statistically significant, and we find an over representation of the Expressives in the non neutral PCs as well.

**Table 1:** For each speech act, the fraction of sentences per press conference labelled with that speech act, grouped by Tightening or Easing and Neutral PCs, plus whether the observed differences are significant according to the $\chi ^2$ test

Speech act	Tightening/Easing	Neutral	$\chi ^{2}$
	($N=4806$)	($N=5502$)	
Declarative	0.04	0.02	$\chi ^{2}(1)=63.05, p < .001$
Assertive	0.52	0.62	$\chi ^{2}(1)=103.65, p < .001$
Modest Directive	0.12	0.09	$\chi ^{2}(1)=16.20, p < .001$
Strong Directives	0.06	0.04	$\chi ^{2}(1)=12.77, p < .001$
Expressive	0.08	0.06	$\chi ^{2}(1)=9.78, p < .01$
Commissive	0.11	0.11	$\chi ^{2}(1)=0.28, p = .6$

**Fig. 6:**
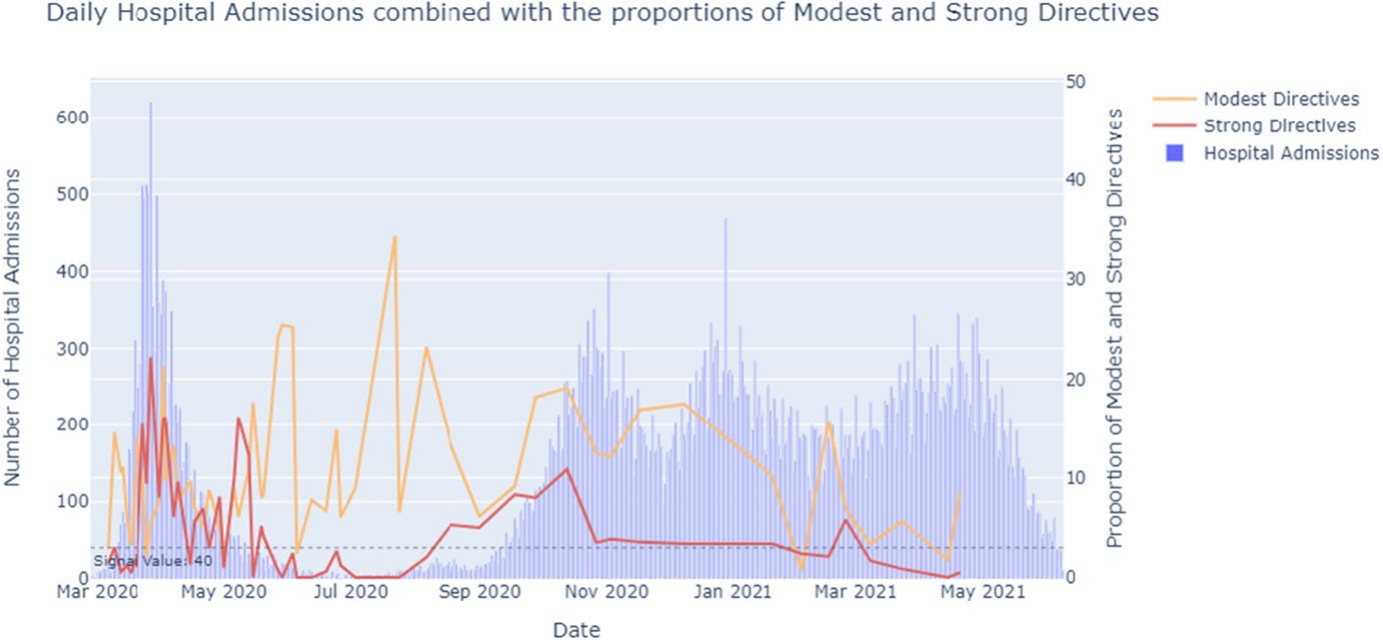
The number of hospital admissions combined with the percentage of Modest and Strong Directives (out of the total 100%) used per press conference

**Fig. 7:**
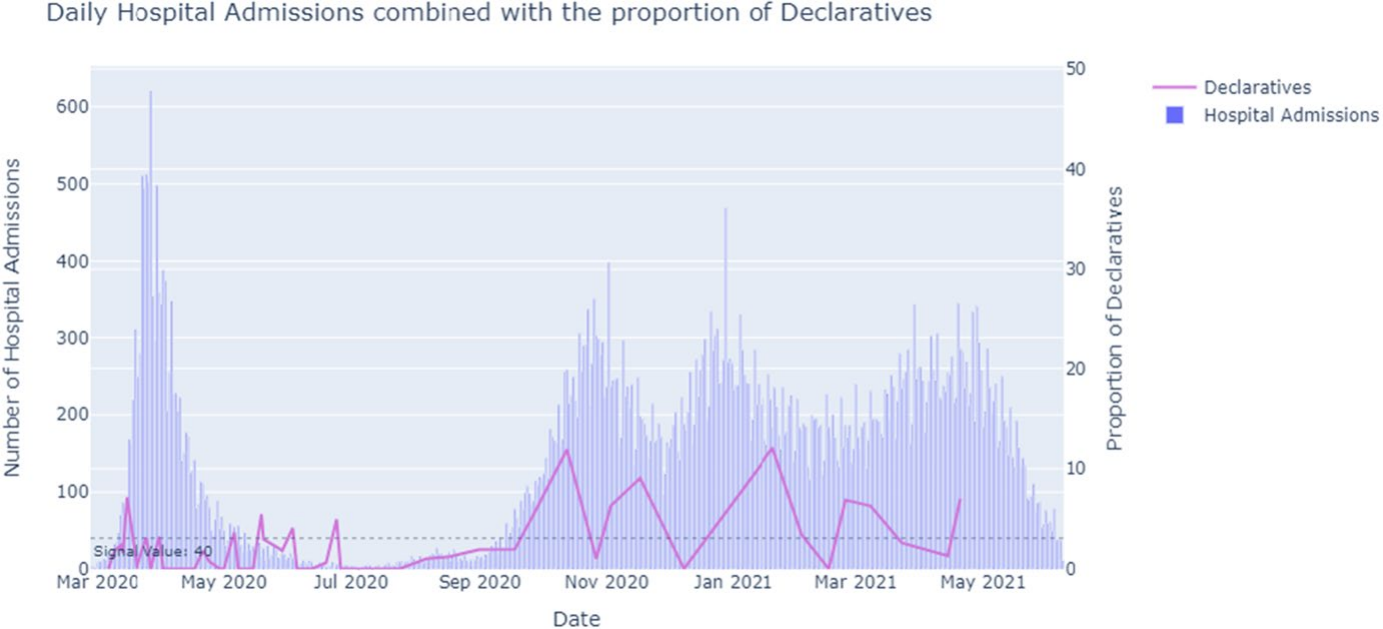
The number of hospital admissions combined with the percentage of Declaratives (out of the total 100%) used per press conference

*Speech Act distribution and the number of daily hospital admissions*In the first peak of Corona hospital admissions (March-April 2020), the press conferences consisted of relatively more Modest and Strong Directives (Fig. 6). Additionally, this period showed tightening press conferences. The first Corona measures were announced, reflected by the Declaratives (Fig. 7). The Dutch were in the so-called Intelligent Lockdown.

During the months May, June and July 2020, the number of hospital admissions reached below the signal value of 40 a day. This period contained easing press conferences. However, the PCs in May still had a notable amount of Directives, with more Strong Directives at first, turning to more Modest Directives as time went on. A possible reasoning for the presence of these Modest Directives could be that the speakers felt the need to keep asking the people to stick to the existing basic main rules despite the easing of measures.

Mid-September 2020, the amount of hospital admissions had risen again, exceeding the signal value of 40 admissions a day. During this period, the proportion of Modest and Strong Directives started to increase as well. In the last weeks of August and the first weeks of September, the governmental representatives tried to steer the people using Expressives and Directives at first. Mid-September, they resorted to additional tightening measures, reflected by the increasing amount of Declaratives.

Mid-November 2020, the amount of hospital admissions started to drop, resulting in an easing press conference on the 17th of November. Shortly after, mid-December, the amount of hospital admissions rose again. On the 14th of December 2020, Prime Minister Mark Rutte held a special speech in ‘Het Torentje’. A strict lockdown was announced. Because this speech is not a press conference, it was not published on the press conferences webpage of the government. Therefore, this speech is not part of the annotated corpus.

During March and April 2021, the amount of hospital admissions rose again, after it had declined slightly during the months January and February. However, on the 14th and 20th of April 2021 measures were eased. These press conferences also show relatively little Modest and Strong Directives. This is against the trend described above. Apparently, other factors were at play in these press conferences.

#### Location of speech acts within a press conference

**Fig. 8:**
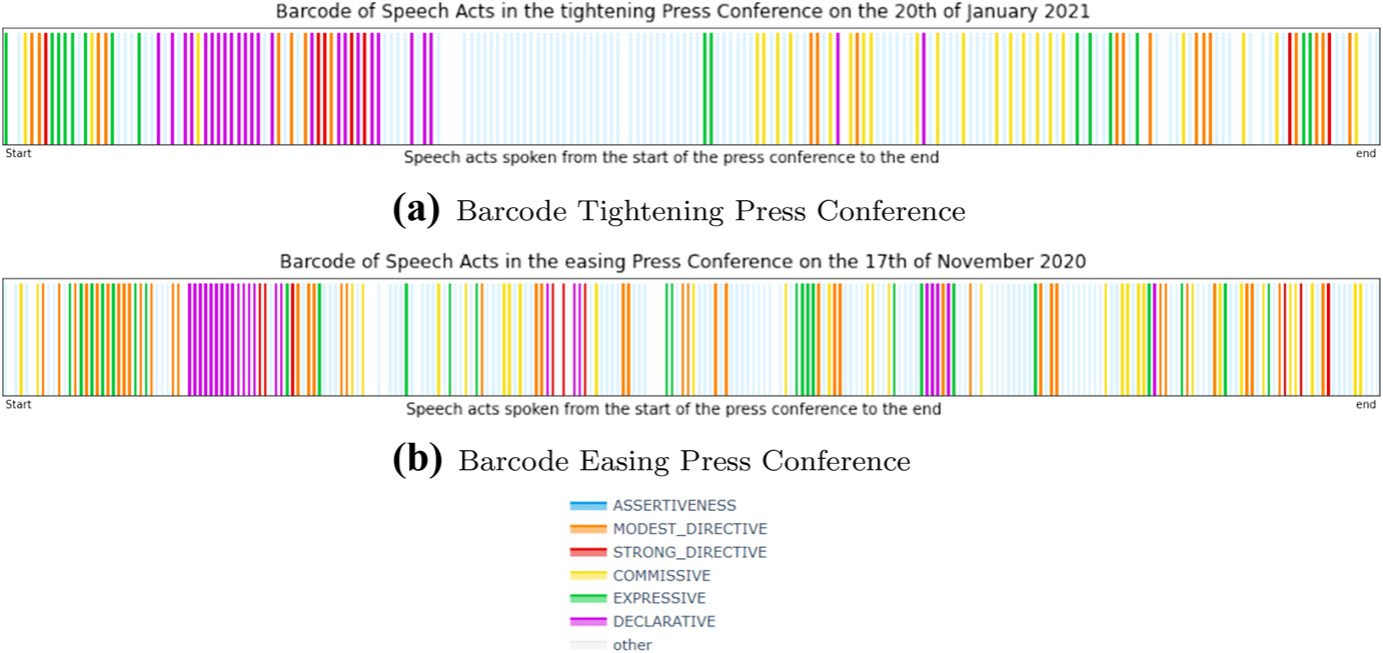
Colored barcodes of the tightening press conference on the 20th of January 2021 and the easing press conference on the 17th of November 2020. Each bar in this graph represents, in chronological order, a sentence spoken in the press conference. The color of the bar indicates the speech act of the sentence, following the same color scheme used previously

We now look whether there are patterns in the *locations* of the speech acts inside the press conferences. To appreciate such a ”close reading” approach, look at Fig. 8, which shows a PC as a sequence of colored bars, each bar representing a sentence, and the colors indicating the different speech acts. Let us first discuss these two prototypical PCs.

**Fig. 9:**
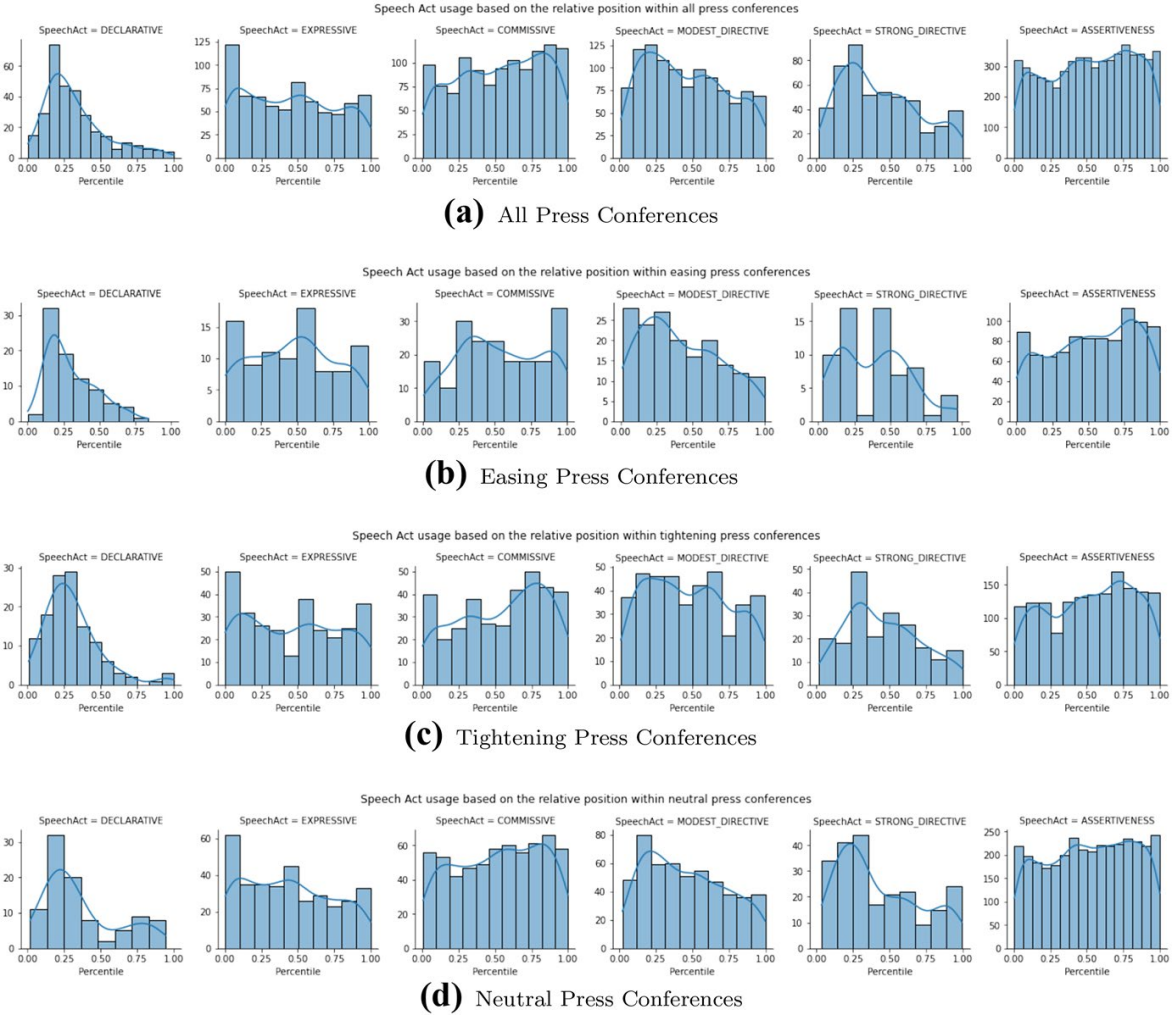
Speech act usage based on their relative position within the press conferences. The horizontal axis indicates the relative position (i.e. percentile) in which the speech act was used. The vertical axis shows the absolute frequency of the speech act being used on that relative position

Now we take a broader view in Fig. 9, with subsets of PCs on the rows, and the 6 speech acts in the columns. Each little plot then depicts the absolute distribution of that speech act in that subset of PCs over the positions in the PC, measured in percentiles. Note that the y-axis scale varies both in the columns and the rows. But instead of the absolute numbers, we focus on the shape of the Kernel Density Estimation (KDE) line. The rows in the figure depict all, the easing, the tightening and the neutral PCs, respectively. What patters can we observe?

First of all, the Declaratives in column one. The population density plots show that the Declaratives are most often present in the first quarter of the press conferences, around the 20th percentile. This is evident in all types of press conferences (all four rows). Thus, Declaratives have a preferred location within a press conference and this location is not influenced by the type of press conference.

Second, the Expressives in column two. The Expressives are mostly present in the beginning of the press conferences, showing a little peak in the middle and in the end. The peak in the middle might relate to the fact that Minister De Jonge starts his introduction halfway. This pattern is evident in all four press conferences. Thus, Expressives are used more often in specific locations. The type of press conference does not influence these locations.

Third, the Commissives. In general, the Commissives are more often present at the end of the press conference. In easing press conferences, Commissives are mostly present in the beginning, around the 30th percentile and they spike at the end of the press conferences during the round of questions. In tightening press conferences, Commissives are mostly present at the end of the press conferences, around the 80th percentile. In neutral press conferences, Commissives do not have a clear location preference. Thus, Commissives have a preferred location, which is influenced by the press conference type.

Fourth, the Modest and Strong Directives. Both types of Directives are mostly present in the beginning of the press conferences. The same pattern holds for easing, tightening and neutral press conferences. Directives and Declaratives are mostly used in the same part of the press conference.

Finally, the Assertives. In general, the Assertives are quite evenly present, but tend to be used more often at the end of the press conferences. They are least present around the 25th percentile, which is also the percentile in which the Commissives and Declaratives tend to be most present. This pattern holds for all press conferences.

The general structure of a tightening press conference can be described as follows: First, some Expressives are used, followed by the Declaratives and the Modest and Strong Directives. These Declaratives and Directives are then explained by the use of Assertives, which are consistently present from this point on. Halfway, additional Expressives are used. In the second half of the press conference, Commissives are used, followed by some more Expressives near the end. The press conference on the 20th of January 2021 is a press conference that shows this general structure. Figure 8a shows a colored barcode of this press conference. Each bar in this graph represents a sentence in the press conference. The color of the bar indicates what speech act the sentence is classified as. The speech acts follow the same color scheme used previously, Assertives being light blue, Expressives being green, Commissives being yellow, Declaratives being purple, Modest Directives being orange, Strong Directives being red. Non-labeled sentences are white. This barcode depicts the general structure described above.

The general structure of an easing press conference is very similar to the general structure of a tightening press conference. The main difference lies in the location of the Commissives. In easing press conferences, the Commissives are more often present in the first half of the press conference. A second color barcode was constructed for the easing press conference on the 17th of November 2020, which is depicted in Fig. 8b. Again, we see the presence of the Expressives in the beginning of the press conference, followed by the Declaratives and the Modest and Strong Directives. Then, these are explained by the Assertives, which are consequently present from this point on. Again, Expressives are used in the middle and at the end.

The difference in Commissives between tightening and easing press conferences is evident when these two barcodes are compared. The easing barcode shows a concentration of Commissives in the second quartile and at the end of the press conference. The tightening barcode shows no Commissives in the second quartile. Instead, the Commissives are concentrated in the third quartile.

#### Difference in speech act usage between Rutte and De Jonge

**Fig. 10:**
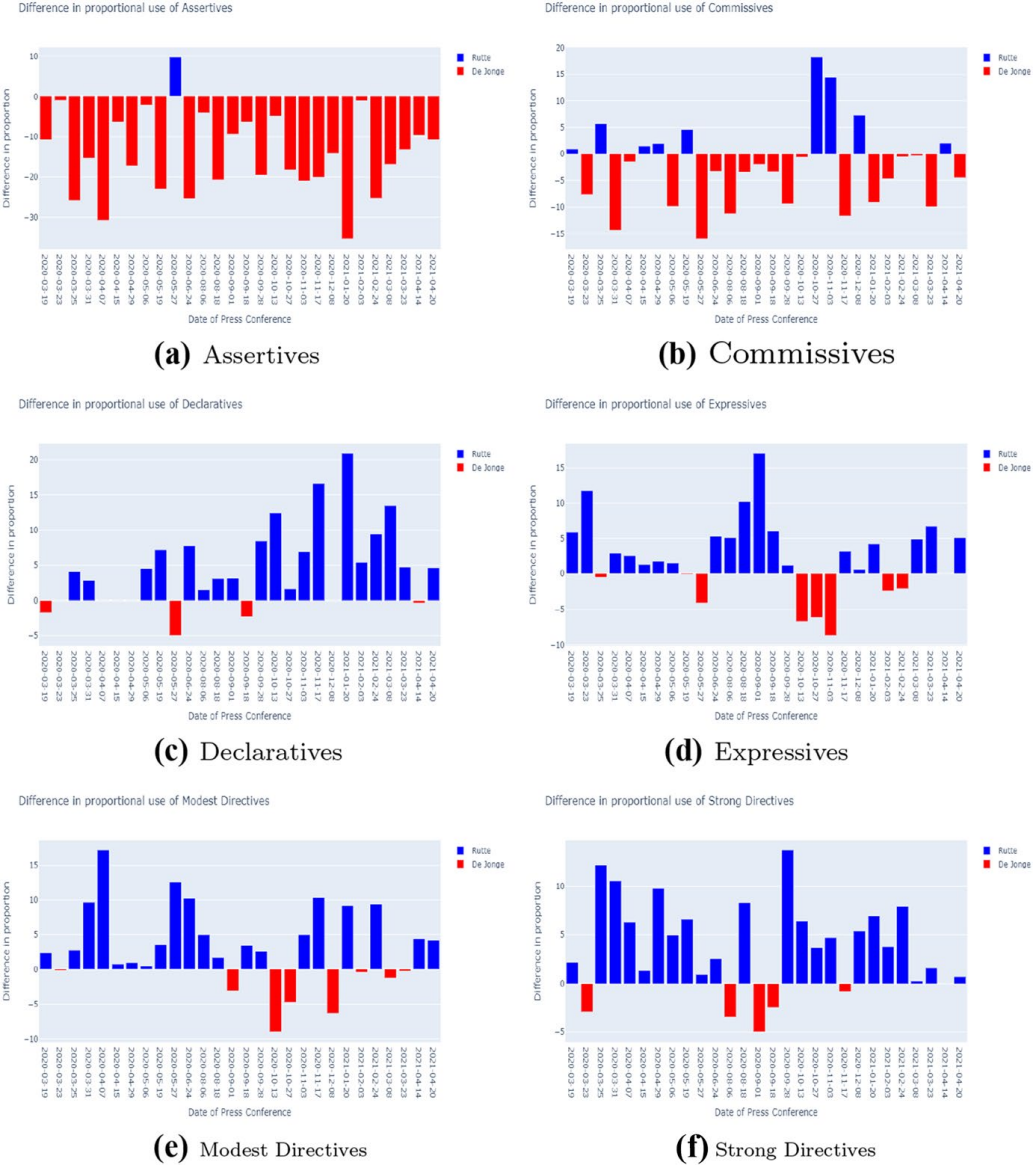
Comparison in speech act usage between Rutte and De Jonge. The amount of speech act annotations was normalized on the total amount of annotations for each speaker. The speech act usage was compared for the press conferences in which both speakers were present. For each of these press conferences, the speech act proportions were subtracted

**Fig. 11:**
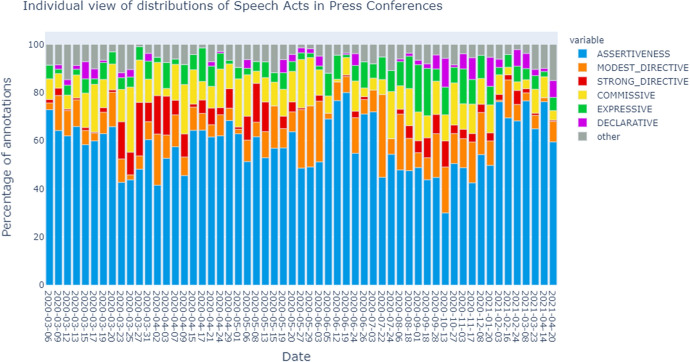
A stacked bar chart showing the speech act distribution per press conference individually. This bar chart enables easy comparison between press conference distributions

In this section, the difference in speech act usage between Prime Minister Mark Rutte and Minister of Health, Welfare and Sport Hugo De Jonge will be analysed. Rutte and De Jonge are the two main governmental representatives in the press conferences. In the press conferences, Rutte and De Jonge do not always speak the same amount of sentences. Therefore, for this comparison in speech act usage between the two speakers, the amount of speech acts annotations was normalized by the total amount of annotations for each speaker. Fig. 10 compares the speech act usage for the PCs in which both speakers were present. For each of these PCs, the speech act *proportions* of de Jonge were subtracted from those of Rutte. Thus a blue (positive) bar indicates overuse by Rutte, and a red (negative) bar overuse by De Jonge.

In Fig. 10a, it is evident that in most press conferences, De Jonge’s proportion of Assertives was higher than Rutte’s. Figure 10b shows that more often, De Jonge’s proportion of Commissives is higher. The graphs on the Expressives, Declaratives, Modest Directives and Strong Directives show that for these speech acts, Rutte’s proportion was more often higher than De Jonge’s.

What can be derived is that De Jonge is often responsible for informing the public on the current state of affairs using Assertives. Additionally, he is often responsible for addressing the government’s future steps in healthcare matters, like testing facilities and vaccination programs using Commissives. This is in line with his function as Minister of Health, Welfare and Sport. Furthermore, Rutte is mainly responsible for announcing regulations, which are reflected by the Declaratives. Additionally, he is responsible for steering the people’s behaviour in the desired direction by using Modest and Strong Directives in combination with Expressives. This is in line with his function as Prime Minister.

### Can machine learning speed up the annotation process?

We did not use machine learning (ML) in the annotation process, but now that we have a large volume of manually labeled sentences, we can address this question. In fact, it is relevant because at the time of writing several new Covid press conferences have occurred in the Netherlands, and we may want to update the created corpus.

The machine learning problem at hand is an instance of what is called *multi-label, multi-class text classification*: we can add a (possibly empty) set of 6 different speech acts to sentences. There are two ways in which we can apply ML: let the algorithm decide on the class (no more human in the loop), or let the algorithm give a ranked list of suggested labels, and let a human pick the correct ones. The first reduces most of the (annotation) costs but with a potential loss in annotation quality. The second will still have substantive annotation costs but with likely no loss in quality.

We will look at both scenarios^1^, and see the influence of the amount of manually labelled training data on the scores. For the first scenario, we simply compute the *accuracy* of the classifier (how often was it correct); for the second we compute the *reciprocal rank* of the correct class (i.e., 1 divided by the rank of the correct class) and take the mean over all speech act classes (macro averaging) or over all sentences in the test set (micro averaging). For simplicity and ease of interpretion of the metrics, we did our experiments on the sentences labeled by zero or at most one speech act (N = 8628)^2^.

We test two text classification approaches. First, a commonly used strong baseline, logistic regression on TF-IDF weighted word uni- and bigrams. Second, a state of the art text classification algorithm based on text embeddings, Roberta, trained on a Dutch corpus (Liu et al. (2019); Delobelle et al. (2020)). Our experimental setup is simple and realistic. We rank all our labelled sentences chronologically. We always use the last 20% as the test set, and vary the training set from the first 20% to the first 80%, in steps of 20%. We used grid search on the training set to find the optimal hyperparameters. All details of the settings of the experiment and more detailed results can be found in the SpeechActClassifier notebook in the dataset repository belonging to this paper.

We summarize our findings. When we look at *accuracy*, the two classification approaches perform almost identical having an almost maximal accuracy already with 40% of the training data. See Table 2. Both classifiers tend to make the same mistake: misclassify one of the five speech acts as an (majority class) Assertive.

**Table 2:** Accuracies for Logistic Regression and Roberta single label speech act classification, with varying amount of training, and testing on the last 20% of the sentences

Train portion	Roberta	LR
0.20	0.65	0.67
0.40	0.72	0.71
0.60	0.72	0.71
0.80	0.73	0.72

The second scenario is evaluated with the mean reciprocal rank. The micro average takes the mean over all sentences, and is dominated by the majority class of Assertives. The macro average takes the average over the mean reciprocal ranks of the 7 classes and is a more meaningful measure given our intended use of the rankings. Table 3 contains the crucial results. We see that both classifiers have the same micro score of 0.83 with 80% of training data, but a quite different macro MRR score (.62 for LR versus .74 for Roberta). The semantically oriented text embeddings classifier is better at classifying the individual classes than LR working with only the lexical information. Table 4 in the Appendix contains a detailed overview of the scores for each speech act. Here we can see that all classes have (much) higher scores with Roberta than with LR, over each amount of training samples, at the cost of the majority class of Assertives. This explains the large increase in macro averaged reciprocal rank, with an equal micro averaged one.

**Table 3:** Micro and Macro Mean Reciprocal Rank for Logistic Regression and Roberta single label speech act classification, with varying amount of training, and testing on the last 20% of the sentences

Train portion	Roberta		LR	
	Micro	Macro	Micro	Macro
0.20	0.80	0.59	0.76	0.74
0.40	0.82	0.59	0.82	0.75
0.60	0.82	0.60	0.82	0.75
0.80	0.83	0.62	0.83	0.74

With Roberta, all speech act classes have an MRR of at least .5, even with only 20% of training data. As the correct class on the first rank yields 1 point and at the second half a point, an MRR above .5 means that on average, the correct class is found in the first two ranks. We can conclude that if high quality labels are desired, a dual annotation system in which an Roberta based algorithm ranks the speech act classes for each sentence and a human corrects the judgement works well and saves annotation time, even with relatively little (N=1725 sentences) training data.

## Conclusion

In this work, the Dutch Corona press conferences between March 2020 and April 2021 have been annotated with and analyzed in terms of Searle’s Speech Act taxonomy. The corpus is made openly available. We briefly recap our main findings.

The created corpus was manually annotated. Based on the inter-rater reliability score, speech acts were identified in a consistent and sufficient way. When identifying speech acts, the use of Assertives in implicit speech acts, the ‘double speech acts’ and the fact that the classification of an utterance can be context dependent are points of attention.

The speech act distribution per PC is related to the type of the PC. Assertives are used more in neutral press conferences than in easing or tightening press conferences. Thus, the focus of neutral press conferences is to inform people on the current state of affairs. The focus of easing and tightening press conferences is on declaring change in regulations, shown by the higher number of Declaratives.

The speech act distribution is also influenced by the number of hospital admissions. In times of high hospital admission numbers, press conferences were tightening and more Modest and Strong Directives were present. In times of low hospital admission numbers during the summer of 2020, press conferences were easing and the presence of Strong Directives reduced over time while the presence of Modest Directives stayed high. This shows that when the number of hospital admissions is high, the speakers are ordering, commanding and insisting the hearers to comply with the regulations, as opposed to asking and pleading when hospital numbers are lower.

The press conferences have a general structure with speech acts having a preferred location within the PC.

The speech acts show that speakers have a distinct role. Rutte, the Prime Minister, is mainly responsible for announcing regulations, which are reflected by his use of Declaratives. Additionally, he is responsible for steering the people’s behaviour in the desired direction by using Modest and Strong Directives in combination with Expressives. De Jonge, the minister of Health, is often responsible for informing the people on the current state of affairs using Assertives. Additionally, he is often responsible for addressing the government’s future steps in healthcare matters, like testing facilities and vaccination programs using Commissives.

Finally, the potential of a machine learning speech act classifier in the context of COVID-19 Press Conferences has been shown. The baseline and the state of the art classifiers scored the same reasonable but not sufficient score of .73 on accuracy. The state of the art scored much better in ranking the speech act classes given a sentence, with the correct class on average on the first or second position (out of 7).

## Supplementary Information

Below is the link to the electronic supplementary material.

Electronic supplementary material 1 (PDF 109 kb)

## Additional figures

See Fig. 11 and Table 4.

**Table 4:** Single label text classification with Roberta and Logistic Regression. Mean Reciprocal Rank for each class plus macro and micro averages with varying amounts of training data, tested on the last 20% of sentences

	20%	40%	60%	80%
ASSERTIVENESS (LR)	0.90	0.94	0.94	0.94
ASSERTIVENESS (Roberta)	0.76	0.85	0.85	0.87
COMMISSIVE (LR)	0.73	0.73	0.72	0.75
COMMISSIVE (Roberta)	0.82	0.83	0.86	0.87
DECLARATIVE (LR)	0.48	0.50	0.48	0.50
DECLARATIVE (Roberta)	0.80	0.78	0.73	0.72
EXPRESSIVE (LR)	0.59	0.52	0.52	0.53
EXPRESSIVE (Roberta)	0.77	0.71	0.75	0.70
MODEST_DIRECTIVE (LR)	0.66	0.66	0.62	0.65
MODEST_DIRECTIVE (Roberta)	0.76	0.75	0.74	0.75
STRONG_DIRECTIVE (LR)	0.38	0.36	0.44	0.48
STRONG_DIRECTIVE (Roberta)	0.54	0.60	0.59	0.59
OTHER (LR)	0.41	0.44	0.48	0.52
OTHER (Roberta)	0.75	0.72	0.70	0.71
Total MRR (Micro) (LR)	0.80	0.82	0.82	0.83
Total MRR (Micro) (Roberta)	0.76	0.82	0.82	0.83
Total MRR (Macro) (LR)	0.59	0.59	0.60	0.62
Total MRR (Macro) (Roberta)	0.74	0.75	0.75	0.74

## Footnotes

1 In fact, we also looked at a third binary classification scenario in which we separated the not very interesting but large (68%) no speech act or assertive class from the union of the five interesting speech act classes. The idea being that if a classifier could do this with a very high recall for the small interesting class, a manual annotator needed only to read and label those sentences. Unfortunately, our best classifier could reach 95% recall only when classifying almost all sentences as the small class, so that would not reduce annotation time.

2 For the multi label case, we can use Jaccard similarity and mean average precision instead of accuracy and MRR, respectively.
