# Supplementary material for: Speech acts in the Dutch COVID-19 Press Conferences
Source: Lang Resour Eval. 2022 Jul 19;57(2):869–92. doi: 10.1007/s10579-022-09602-7 (PMC9294846; doi:10.1007/s10579-022-09602-7)
Supplement: Supplementary file 1 — Electronic supplementary material 1 (PDF 109 kb) [file 10579_2022_9602_MOESM1_ESM.pdf]

Annotation Guidelines

Annotating protocol Speech Acts in Dutch COVID-19 Press Conferences

General guidelines on how to annotate

These annotation protocols are focused on Searle's five taxonomies of speech acts. Searle states that the purpose of language is to do things. A speech act is an utterance that is considered as an action. It serves a function in communication. With speech acts, Searle provides a universal characterization between language use and actions. Searle defines five categories of speech acts: Assertives, Directives, Commissives, Expressives and Declaratives. Directives can either be modest or strong. Therefore, these protocol guidelines also prescribe the distinction between modest and strong directives. Speech acts can be distinguished not only by explicit text, but also by implicit meaning derived from the context and setting.

Example of an explicit expressive:

- *<u>We zijn natuurlijk blij</u> dat dit nu kan, want de samenleving snakt naar meer ruimte.*

Here, the speaker directly states its psychological attitude towards the state of affairs.

Example of an implicit expressive:

- *Dat maakt ons besluit van vandaag ook zo ongelooflijk spannend*

Here, the speaker does not directly state 'I am anxious about this decision' but from this sentence can be derived that he is.

General Annotation Instructions
The annotation process takes place in Prodigy. You will be shown an individual sentence. Select applicable speech acts by clicking the tick boxes. If you have selected the applicable speech acts, press the green button to accept the sentence and confirm the selected speech acts.

If a sentence falls in more than one category, select all speech acts that are applicable.
An example where an utterance falls into multiple speech act categories:

- *Dat zijn we verplicht aan alle mensen in de zorg.*

This sentence is a Modest Directive as well as an Expressive. Here the speaker pleads with the hearer to do something and at the same time expresses his feeling of responsibility and solidarity.

If no speech act is applicable, select nothing and click the green button to accept the sentence.

If the sentence shown is split incorrectly and therefore displaying more than one sentence or if the sentence shown is unreadable due to parsing errors, press the red button to reject the sentence. For example:

- *En voor zalen met een groot cultureel belang geldt dat voorzitters van de veiligheidsregio's ook een uitzondering **makenEen** volgend blok maatregelen heeft betrekking op wat we met een lelijk woord doorstroomlocaties zijn gaan noemen.*

Annotate the sentences as they are read individually, without carrying the knowledge of the previous sentence into the current one.

Annotation protocol Assertives (ASS)

The aim of this protocol is to annotate sentences that contain Assertive Speech Acts.

Assertives: The speaker wants to commit the hearer to his belief on how things are in the world. The speaker says something is being the case, or will state a representation of reality. This statement can be found true or false.

Key words: statements, descriptions, classifications, explanations, clarifications, assertions, claims, hypothesis, suggestions.

An example of a sentence that is an Assertive speech act:

- *De instroom en het aantal corona patiënten in de ziekenhuizen vlakken nu af.*
- *Het Europees Medicijn Agentschap heeft daar goed naar gekeken en is op basis van de nu gerapporteerde meldingen, tot de conclusie gekomen dat die mogelijke bijwerking weliswaar op zou kunnen treden, maar dat het aantal gemelde gevallen extreem laag is.*

Annotation protocol Directives (MDIR & SDIR)

The aim of this protocol is to annotate sentences that contain Directive Speech Acts.

Directives: These are attempts by the speaker to try to get the hearer to do something. The speaker aims to make someone else do something he desires. Directives refer to future acts. Directives are obeyed or disobeyed. Directives can be either modest or strong.

Key words modest directives: inviting, advising, requesting, asking, pleading, begging.

Key words strong directives: ordering, commanding, insisting.

An example of a sentence that is a Modest Directive speech act:

- *Ik doe een beroep op mijn landgenoten, uw landgenoten, ons aller landgenoten om hier verstandig mee om te gaan in volle solidariteit met mensen die ziek zijn en de mensen die zo vreselijk hard werken in de ziekenhuizen*
- *Daarom roep ik iedereen op om wat vaker 's ochtends de boodschappen te doen, want dan is het een stuk rustiger in de winkels.*

An example of a sentence that is a Strong Directive speech act:

- *En wat hier staat blijft gelden, je handen wassen, afstand houden, testen bij klachten.*
- *Die mensen, die dus voor hun werk op pad zijn, moeten dan ook een werkgeversverklaring bij zich hebben.*

An example of an explicit directive:

- *Blijf thuis!*

An example of an implicit directive:

- *Kortom, een tijd waarin we ook weer willen benadrukken, ook op de scholen, hoe belangrijk het is dat zieke kinderen niet naar school gaan.*

Annotation protocol Commissives

The aim of this protocol is to annotate sentences that contain Commissive Speech Acts.

Commissives: Commissives commit the speaker to some future course of action. It shows the intention of the speaker in the future.

Key words: promises, offering, oaths, pledges, threats, vows, guarantees, covenants.

An example of a sentence that is a Commissive speech act:

- *Ik beloof u dat ik u steeds zal vertellen wat er in mijn hoofd zit, wat we weten, wat we niet weten en op basis waarvan we die afwegingen maken.*
- *En wie er niet aan voldoet en bijvoorbeeld toch klanten toelaat in de winkel, die wordt gesloten.*

An example of explicit commissive

- *De komende maanden gaan we nog veel meer en veel vaker testen.*
- *Het kabinet houdt - zoals steeds - de vinger aan de pols met de steunpakketten.*

An example of implicit commissive

- *En we gaan er vooralsnog vanuit dat dit vanaf 1 juni kan.*

Annotation protocol Expressives

The aim of this protocol is to annotate sentences that contain Expressive Speech Acts.

Expressives: These express the psychological attitudes and emotions of the speaker towards the state of affairs. They state what the speaker feels.

Key words: thanking, apologizing, congratulating, welcoming, greeting.

An example of a sentence that is a Expressive speech act:

- *En daar heb ik ook zelf de afgelopen dagen enorm mee geworsteld.*

Example of an explicit expressive:

- *<u>We zijn natuurlijk blij</u> dat dit nu kan, want de samenleving snakt naar meer ruimte.*

Here, the speaker directly states its psychological attitude towards the state of affairs.

Example of an implicit expressive:

- *Dat maakt ons besluit van vandaag ook zo ongelooflijk spannend*

Here, the speaker does not directly state ‘I am anxious about this decision’ but from this sentence it can be derived that he is anxious.

Annotation protocol Declaratives

The aim of this protocol is to annotate sentences that contain Declaratives Speech Acts.

Declaratives: These speech acts change the world by verbally stating the change. The speaker must have some contextual privileges that allow him or her to declare this change. The saying is the doing. To test if something is a declarative utterance, you can add the word ‘hereby’ in front of it. For example: I (hereby) pronounce you husband and wife.

Key words: hereby, declaring, approving, blessing, dismissing.

An example of a sentence that is a Declaratives speech act:

- *Vanaf woensdag 28 april mogen de buitenterrassen onder voorwaarden weer open van 12 tot 6 uur s ’middags.*

An overview:

**Speech Act**	**Relation**
Assertive	Speaker believes Situation
Modest Directive	Speaker wants Situation
Strong Directive	Speaker demands Situation
Commissive	Speaker intends Situation
Expressive	Speaker feels Situation
Declarative	Speaker causes Situation

REFERENCE

- Nicholas Fotion. (2000). *John Searle.* Routledge.
- Searle, J. R. (1979). *Expression and meaning: Studies in the Theory of Speech Acts.* Cambridge University Press.
- Smith, B. (2003). *John Searle.* Cambridge University Press.
